# Experiences of caregivers of children with cancer in Malawi: A qualitative study

**DOI:** 10.1002/cam4.6963

**Published:** 2024-02-06

**Authors:** Lophina Phiri, William Ho Cheung Li, Patrick G. M. C. Phiri, Ankie Tan Cheung, Watipaso Wanda‐Kalizang'oma, Anizia Kamwendo, Sellina Lemon

**Affiliations:** ^1^ The Nethersole School of Nursing, Faculty of Medicine The Chinese University of Hong Kong Shatin New Territories Hong Kong; ^2^ Institute of Applied Technology, Fatima College of Health Sciences Al Ain Abu Dhabi United Arab Emirates; ^3^ Baylor College of Medicine Childrens Foundation, Global HOPE Project Lilongwe Malawi; ^4^ Queen Elizabeth Central Hospital Blantyre Malawi

**Keywords:** cancer, caregiver, child, experience, low‐income country, qualitative study

## Abstract

**Background:**

Studies have shown that caregivers of children with cancer experience challenges when caring for their children. To date, no studies have examined the experience of caregivers of children with cancer in Malawi, a low‐income country in sub‐Saharan Africa. Hence, this study aimed to explore the experiences of caregivers of Malawian children receiving cancer treatment.

**Methods:**

This explorative qualitative study used semi‐structured interviews to collect data from 22 caregivers of children receiving cancer treatment. The data were analysed using qualitative content analysis.

**Results:**

Five themes emerged from the QCA. The caregivers perceived their children's cancer as a burden, a form of psychological torture and a disruptor of family routines and social lifestyles. They also reported self‐isolation due to the stigma that they faced in the course of caring for their children and a need for informational, psychosocial, spiritual and financial support.

**Conclusion:**

Caregivers of Malawian children with cancer experience physical and psychosocial challenges as they are caring for their children with cancer. Developing appropriate interventions would enable nurses to offer optimal support to these caregivers in dealing with these challenges and meeting their needs effectively.

## 1 INTRODUCTION AND BACKGROUND

Globally, the World Health Organisation (WHO) estimates that around 400,000 children aged 0–19 years are diagnosed with cancer annually.^1^ The incidence of childhood cancer varies significantly between regions, with approximately 90% of the cases being from low‐ and middle‐income countries (LMICs), where 95% of children of this age live.^1^ In Africa, cancer incidence is particularly high, with an estimated 100,000 new cases annually under the age of 15 years and the incidence is expected to double by 2030.^2^, ^3^ Malawi, like other LMICs in sub‐Saharan Africa, has a high childhood cancer burden, with 1108 cases reported in 2020.^4^ Acute lymphoblastic leukaemia is the most common childhood cancer globally, followed by non‐Hodgkin lymphoma, nephroblastoma, Burkitt lymphoma and Retinoblastoma.^1^ In Africa, the most common cancer in children is lymphoma, retinoblastoma and renal tumours,^1^ while In Malawi, acute lymphoblastic leukaemia was reported as the most common cancer in 2020.^1^ While the survival rates for paediatric cancer have improved significantly due to advancements in treatment and supportive care,^5^ they remain significantly lower in LMICs compared with high‐income countries, with an estimation of an 80% survival rate in high‐income countries and less than 30% in LMICs.^1^

The diagnosis of cancer in a child impacts the child's family and disrupts its routines and structures. In addition, childhood cancer treatment negatively affects caregivers' physical, social, and psychological well‐being and their professional routines, as they must pay complete attention to their child with cancer for prolonged periods.^6^, ^7^ The most common feelings experienced by caregivers include shock, despair, fear, guilt, loss, denial and anxiety.^8^, ^9^ These feelings arise due to caregivers' uncertainty about their child's prognosis and fear of their child's death. Caregivers often feel helpless, abandoned and lonely, as they must leave their homes and devote their time to caring for their ill child.^10^ Furthermore, parents may experience financial challenges due to the costs of travelling between their home and hospital and the need to pay for accommodation and food during their child's treatment in hospital.^11^

In Malawi, caregivers are essential in supporting their children in hospitals and at home.^12^ Moreover, caregivers provide physical and emotional support for their children.^13^ However, these caregivers experience several challenges during cancer treatment. There are limited resources, such as few trained paediatric oncology doctors and nurses, insufficient cancer diagnostic capacity and infrastructure, and a shortage of essential chemotherapy, just as in other low‐income countries.^14^ Additionally, the patients often report late for treatment, which affects the prognosis of treatment since they come when cancer is at an advanced stage, a situation that leads to a loss of hope in many caregivers.^15^ Initially, most caregivers do not suspect cancer at first; they first suspect witchcraft and visit the traditional healers before going to the hospital. Others may visit several other health providers and hospitals, such as dispensaries, health centres, private clinics and district hospitals before cancer is diagnosed.^16^ Once the diagnosis is made, the caregivers fear that cancer may recur when the treatment is finished, or the child will die. As a result, the caregivers may be predisposed to physical, psychological and social challenges, including fear, shock, grief and isolation.

Treatment abandonment is a prevalent issue in sub‐Saharan Africa, and Malawi is no exception.^15^, ^17^, ^18^ One of the primary reasons for treatment abandonment among caregivers of Malawian children with cancer is the lack of knowledge about cancer and its treatment. This lack of knowledge is also evident at the community level, where the caregivers are advised to stop treatment and look for alternative therapies, such as traditional healers, due to the belief that the child's illness is supernatural.^15^ Another significant challenge is that long‐distance caregivers and patients must travel to access treatment. Sadly, poor road infrastructure exacerbates this issue, making it challenging to reach facilities. In Malawi, the Specialised higher‐level diagnosis and treatment for cancer patients is available in only two referral hospitals: Kamuzu Central Hospital in Lilongwe and Queen Elizabeth Central Hospital in Blantyre. Stanley et al. in their study, caregivers cited high transport costs as a challenge despite receiving the transport reimbursement.^15^ This, coupled with the high cost of living and poverty, means long hospital stays can cause significant financial strain on caregivers, predisposing caregivers to more financial strain.

Understanding the experience of caregivers of children with cancer is essential for the provision of tailored support.^19^ Moreover, by examining the caregivers' experiences during their children's treatment, interventions can be developed to support their caregiving experience. While the experiences of caregivers of children with cancer have been studied in other countries,^20^, ^21^, ^22^, ^23^ little is known about the experiences of caregivers of children receiving cancer treatment in Malawi. The caregiving experience can be influenced by cultural, social and economic factors specific to each country. Thus, conducting this study in Malawi provides insight into the unique experiences of caregivers in that particular context. This study aimed to explore the experiences of caregivers of children receiving cancer treatment in Malawi.

## 2 METHODS

### 2.1 Study design

This study used a qualitative exploratory research design involving 22 caregivers to explore the experience of caregivers of children with cancer. Qualitative research provides a multi‐dimensional and in‐depth exploration and analysis of the phenomenon being studied, allowing participants to share their perspectives and real‐world experiences.^24^

### 2.2 Study setting and participants

Participants were selected from the paediatric oncology units of Queen Elizabeth Central Hospital (QECH) and Kamuzu Central Hospital (KCH), the major paediatric cancer centres in Malawi. The study enrolled caregivers of children who were diagnosed with cancer within 1 year and receiving treatment. Purposive sampling was employed to identify and recruit suitable study participants.

### 2.3 Data collection procedure

Data were collected from individual participants through face‐to‐face, in‐depth interviews between December 2022 and January 2023. After obtaining written consent, the interviewer collected sociodemographic data by asking the participants and reviewing the children's medical records. Two nursing researchers with a bachelor's degree in nursing science and experience in qualitative research conducted interviews using a semi‐structured interview guide. Interviews continued until data saturation was achieved after the 22nd participant. All interviews were conducted in a private room in the paediatric oncology ward to ensure the participants' privacy. Each interview was audio‐recorded and lasted ~ 20–30 min. Data were secured using a password‐protected computer drive, and participants were identified using numbers. The interviews were conducted in Chichewa and then translated into English by the first author and validated by the third author. The following are the interview questions:

1. What is the initial experience of having a child with cancer?
2. How has cancer affected you physically, psychologically, and socially?
3. What challenges do you face caring for your child with cancer?
4. How do you cope with the stressors/challenges of your child's cancer diagnosis?
5. What support have you received since your child was diagnosed with cancer?
6. What physical/psychosocial supportive care need do you have when caring for your child with cancer?
7. What kind of support do you think is essential to help you cope with childcare challenges?

### 2.4 Trustworthiness

The trustworthiness of this study was achieved through different strategies to achieve credibility, dependability, conformability and transferability.^25^ The credibility was achieved by following the study methodology to ensure congruency between the research methods and study questions. Furthermore, the researchers recruited participants with different demographic characteristics and clinical data. A detailed research process has been provided to achieve the dependability and conformability of this study.^26^ Additionally, to achieve transferability, the researcher has provided a detailed description of the participants' characteristics, site and the data collection procedures.^27^ Field notes and reflexive journals were documented throughout the data process.

### 2.5 Ethical considerations

The current study was approved by the Survey and Behavioural Ethics Committee of the Chinese University of Hong Kong (SBRE), Hong Kong (approval no. SBRE‐21‐0766) and the National Health Science and Research Ethics Committee, Malawi (approval no. 2747). Permission to conduct the study was also obtained from the study sites. Those who expressed interest were given information about the study, assured about their privacy, confidentiality and anonymity, and informed about their voluntary participation without censure. Those who agreed to participate signed consent forms.

### 2.6 Data analysis

The steps of qualitative content analysis (QCA), as suggested by Graenheim & Lundman,^27^ were followed during the data analysis, including transcription of data, identification of meaning unit, condensation of data, grouping data into codes, creating categories and developing themes. Transcription verbatim of the recorded interviews was conducted. The transcripts were reviewed many times to ensure a thorough understanding of the data, allowing for complete immersion in the content and an understanding of the overall context. The next step was identifying and grouping the words and statements that shared a context to form the meaning units. This was followed by data condensation, whereby the researcher condensed the raw data by summarising the data material to produce the condensed meaning units. Then, the condensed meaning units were grouped based on their similarities and given codes. This approach of creating codes facilitated understanding of information in a new and different way. After creating codes, the researcher compared various codes and organised them based on their similarities and differences, ultimately sorting them into distinct categories. The final step was developing the themes based on the categories. The data are reported per the Consolidated Criteria for Reporting Qualitative Research checklist.

## 3 RESULTS

### 3.1 Sample characteristics

Twenty‐two caregivers of children receiving cancer treatment participated in the interviews. The mean age of the participants was 36.96 years [standard deviation (SD) = 10.68], the mean age of children was 8.32 years (SD = 5.18), and the mean duration since child cancer diagnosis was 17.41 weeks (SD = 13.01). The most common cancer was lymphoma (31.82%). Table 1 presents the characteristics of the participants.

**TABLE 1:** Characteristics of caregivers and children with cancer.

	Frequency (%)
Caregivers age	
<31	5.00 (22.73)
31–40	10.00 (45.45)
>40	7.00 (31.82)
Caregiver gender	
Male	5.00 (22.73)
Female	17.00 (77.27)
Marital status	
Married	17.00 (77.27)
Separated	5.00 (22.73)
Ethnic group	
Chewa	6.00 (27.27)
Tumbuka	5.00 (22.73)
Yao	4.00 (18.18)
Lhomwe	4.00 (18.18)
Other	3.00 (13.64)
Religion	
CCAP	3.00 (13.64)
Roman Catholic	6.00 (27.27)
Pentecostal	4.00 (18.18)
Moslem	5.00 (22.73)
Other	4.00 (18.18)
Education	
No education	3.00 (13.64)
Primary	14.00 (63.64)
Secondary	3.00 (13.64)
Tertiary	2.00 (9.09)
Employment status	
Employed	2.00 (9.09)
Business	8.00 (36.36)
Not employed	2.00 (9.09)
Subsistence farming	10.00 (45.45)
Relationship with the child	
Mother	13.00 (59.09)
Father	5.00 (22.73)
Grandmother	3.00 (13.64)
Other relations	1.00 (4.55)
Child age	
1–5	9.00 (40.91)
6–10	4.00 (18.18)
>10	9.00 (40.91)
Child gender	
Male	10.00 (45.45)
Female	12.00 (54.55)
Type of cancer	
Leukaemia	6.00 (27.27)
Lymphoma	7.00 (31.82)
Retinoblastoma	3.00 (13.64)
Wilms tumour	3.00 (13.64)
Other	3.00 (13.64)
Duration of illness	
1–26 weeks	18.00 (81.82)
>26 weeks	4.00 (18.18)

### 3.2 Themes and subthemes identified in the QCA

The QCA identified the following five key themes: (1) Perception of cancer, (2) Inadequate support, (3) Coping strategies and (4) Caregivers' needs. Themes and sub‐themes are presented in Table 2.

**TABLE 2:** Summary of the themes, sub‐themes and categories from the qualitative content data analysis.

Theme	Sub‐themes
Perception of cancer	A physical burden
	A psychological torture
	A disruptor of family routines and social life
Inadequate support	Abandonment by family members
	Isolation and disease‐related stigma
Coping strategies	Information support
	Religious beliefs and practices
	Acceptance
	Social support
	Distractive activities
Caregivers needs	Information needs
	Psycho‐social needs
	Spiritual needs
	Financial and material needs

### 3.3 Perception of cancer

The participants had different perceptions about cancer. Thus, the subthemes were a family burden, psychological torture and a disruptor of family routines and social life.

#### 3.3.1 A physical burden

Most participants (*n* = 13) described cancer care provision as burdensome, overwhelming and tiring. They spent much of their time caring for their child and could not meet their own needs and the needs of their other children, which meant that they were physically overtaxed. In addition, they also experienced a lack of sleep. One participant stated: ‘*He (the child) would cry the whole night, and I could not sleep. He would generally wake at around 10 p.m. and start screaming; this would continue until 2 a.m., and I couldn't sleep because I was trying to calm him’*. (Participant 21).

#### 3.3.2 A psychological torture

Most of the participants (*n* = 20) reported that they were affected psychologically by their child's cancer diagnosis and caregiving responsibilities. Participants felt worried, overwhelmed, saddened, disappointed, frustrated, hurt, upset, hopeless and helpless upon hearing that their child had cancer and as treatment continued. These feelings were due to their inadequate knowledge of cancer and its treatment, uncertainty about whether treatment would be curative, prognosis, survival and a fear that their child might die. The psychological turmoil continued as the child's treatments continued. One participant stated:

I am disappointed and frustrated because I have unanswered questions about this disease. No one has ever explained it to me. Another thing that's troubling me is whether the tumour in my child's body will go away or not. I am worried that even if it goes away, it might come back. I have a lot of thoughts and concerns about whether this disease will ever end or not. (Participant 11).

#### 3.3.3 A disruptor of family routines and social life

Some participants (*n* = 5) stated that cancer caregiving significantly disrupted family routines. As a result, they could not carry out their usual activities, such as running a business or farming to make a living. Additionally, these participants struggled to care for their other children and meet their needs. Moreover, the responsibility of looking after their sick child impacted their relationships. One of the participants said: ‘*I am not at home, so some things I can only do at home cannot be done anymore. This shows how the illness has affected things’* (Participant 18).

### 3.4 Inadequate support

The participants reported lacking social support from family members and the community. Two sub‐themes emerged, including abandonment by family members and self‐isolation due to the stigma associated with their child's cancer diagnosis.

#### 3.4.1 Abandonment by family members

Due to their child's illness being prolonged and thus requiring long‐term management and hospitalisation, some of the participants (*n* = 5) reported having been abandoned by their relatives during hospital stays. These participants felt unsupported emotionally and did not receive any financial and material assistance. This lack of support made it challenging for them to cope with the reality of their child's illness. One participant said:

Other people rushed to help during the early days of the child's illness, but after we had stayed in the hospital for a long time, they all stopped coming and did not even send us anything supportive (Participant 20).

#### 3.4.2 Isolation and disease‐related stigma

Some participants (*n* = 4) had disengaged purposefully from social interactions with friends, relatives, and other family members. They had typically done so to avoid stigma and being in contact with people who spoke negatively about their child's cancer diagnosis, as this increased their levels of stress. One participant said:

I choose to be alone because I do not particularly want people to tell me bad things about my child's illness, as this would make me worry more than I do already. So, I do not associate with people (Participant 7).

### 3.5 Coping strategies

The participants used various coping strategies to cope with stressors associated with their child's diagnosis and caregiving. These coping strategies included informational support, religious beliefs and practices, acceptance, social support and distractive activities.

#### 3.5.1 Informational support

Some of the participants (*n* = 4) reported that the information they had received from healthcare workers (HCWs) and other caregivers of children with cancer had helped to reduce their stress levels. In particular, the information they had received about the cause of their child's cancer, its treatment and its prognosis had helped to decrease their uncertainty about their child's condition. They reported that after receiving the information, they felt more informed and better equipped to cope with the challenges of caring for a child with cancer than before. One participant said:

When my child was diagnosed with cancer, the people at the office where we get medication taught me both positive and negative things about it. They also taught me that cancer can be cured. This information encouraged me and removed my fears (Participant 20).

#### 3.5.2 Religious beliefs and practices

Religious practices were an important coping strategy for most participants (*n* = 12) and involved prayer, reading the Bible, attending church services and visiting religious leaders for prayers. These participants believed that while doctors do their part, God ultimately heals a patient. This belief gave them hope, encouragement and a sense of purpose, which helped to reduce their anxieties. One participant said: *‘I usually go in for prayers. These prayers help me to forget my worries, and I leave everything in the hands of God’* (Participant 14).

#### 3.5.3 Acceptance

Some participants (*n* = 10) reported that they had come to accept the reality of their child's condition. They understood that their child's condition would not change quickly and had decided to ‘move forward’ and help their children receive treatment, which they hoped would help their children get better. One participant said, *‘I have accepted my child's condition, in anticipation that the treatment will help my child to get better. There is nothing else I can do’* (Participant 13).

#### 3.5.4 Social support

Some participants (*n* = 7) reported that social support was an effective coping strategy. These participants received encouragement and emotional support from friends, religious groups and HCWs, which gave them hope and meaning in life. Additionally, they received material support from extended family members, religious groups and hospitals that helped lessen the burden of caring for their ill child. Furthermore, some participants (*n* = 3) reported that interacting with fellow caregivers whose children were responding well to treatment provided encouragement and hope. One participant said:

I receive emotional support from my friends. They normally call me every day and encourage me. Even other caregivers of children with cancer provide support; we support each other through sharing experiences and information (Participant 1).

#### 3.5.5 Distracting activities

Some participants (*n* = 12) engaged in various activities to distract themselves from their worries. These activities included walking, chatting with friends, watching television, watching football, listening to their favourite songs and dancing with their children. One participant said, *‘I walk with my child and chart with him and other friends to take my mind off my worries’* (Participant 13).

### 3.6 Caregivers needs

This theme was characterised by various needs that the participants reported, including informational, psychosocial, spiritual, financial and material needs.

#### 3.6.1 Informational needs

Most of the participants (*n* = 13) expressed that they lacked cancer information, which made it difficult for them to have a clear picture of their child's condition. These participants expressed a need for information on child cancer and related topics, such as information on the prevention, causes and treatment of cancer in children, including the potential side effects of chemotherapy; details on how to care for a child with cancer and nutrition for a child with cancer. Furthermore, they needed information on coping strategies to alleviate stress. One participant said:

I want to know what cancer is, how it starts and how to prevent it. It was explained to me but not in detail − I need to know If there are ways to avoid it; maybe we can prevent cancer. I wish I had been taught this (Participant 21).

#### 3.6.2 Psychosocial needs

Some participants (*n* = 6) reported that it was crucial for their psychological and social well‐being needs to be met. They emphasised the importance of receiving social and psychological support from family members, other caregivers, religious leaders and HCWs. They stated that they would benefit from psychological support such as encouragement, empathy, interaction and sharing of experiences with other caregivers of children with cancer. They also recommended having constructive interaction with HCWs. One participant said:

‘Having people around me to whom I can talk would help me to ‘put away’ my thoughts and worries about my child's illness. HCWs need to provide help to us to deal with our worries caused by the child's illness’ (Participant 2).

#### 3.6.3 Spiritual needs

The participants reported that their religious beliefs provided them with comfort that helped them to cope with their child's illness. However, some participants (*n* = 11) mentioned that they faced challenges in praying alone and suggested the formation of prayer groups in the hospital to support each other. They also recommended establishing designated places for prayers, organising a prayer schedule and spiritual guidance from the hospital chaplain, which they felt would encourage them to pray and strengthen their faith in God. One participant said:

Just make a schedule for prayers. We have other women who know how to share the word of God; they can do that. And even women who do not pray may be helped by hearing the word of God. (Participant 12).

#### 3.6.4 Financial and material needs

The participants expressed a strong need for financial and material support during hospital stays and at home. Many of them reported that their caregiving responsibilities had negatively impacted their source of income. Consequently, they struggled to meet the financial and material needs required to care for their child and other family members. The frequent hospital visits for treatment and follow‐up added to their financial burden, thereby increasing the difficulty they experienced in managing their resources. One participant said:

When caring for a child, we need money, food, or some materials to provide satisfactory care, but we do not have enough. We cannot work or run a business, as we are at the hospital with our children most of the time. We need people to help us meet our financial and material needs (Participant 5).

## 4 DISCUSSION

This study explored the experience of caregivers of Malawian children with cancer receiving treatment. The participants in this study viewed cancer care provision as burdensome, overwhelming and tiring, leading to exhaustion. This is in line with the previous studies, which have reported that cancer diagnosis brought about an extensive increase in the caregivers' roles and responsibilities.^9^, ^28^ Caregiving requires physical effort, and when caregivers have to combine caring roles with other responsibilities such as house chores, work, and looking after other siblings, it can be very demanding and exhausting.^28^ Extreme exhaustion may result in physical challenges to the caregivers, which may interfere with the caregiving process and lead to physical health problems.^28^ This underscores the need for oncology healthcare professionals to ensure that caregivers receive the appropriate resources and support from families, friends and healthcare professionals to care for their children at the hospital and home.

The findings showed that participants in this study reported being worried, saddened, disappointed, frustrated, hurt, upset, hopeless and helpless associated with the news of the child's cancer diagnosis, which continued when the child was getting the treatment. These results confirm the findings of our cross‐sectional study that caregivers of children with cancer in this population experience anxiety and depressive symptoms during the child cancer treatment.^29^ The most common concerns for the caregivers were a lack of knowledge and uncertainty about the prognosis and cure. This finding concurs with previous studies in which caregivers reported negative reactions during the active treatment phase.^23^, ^30^ For example, Tan et al. reported that caregivers experienced shock, confusion and grief; however, the caregivers came to the acceptance of the child's diagnosis.^9^ The life‐threatening nature of the disease is one of the major concerns of most caregivers, especially the sense of imminent death^23^ and cancer is considered a deadly disease with low survival rates.^31^ However, in the current setting, the myths and misconceptions associated with cancer are most profound, which may affect the perception of cancer by most caregivers.

The results of this study also showed that the participants experienced disruption to their routines and social life and concurred with the previous studies on caregivers of children with cancer.^8^, ^23^ Cancer treatment often involves frequent visits to and lengthy stays in hospital and numerous other medical appointments. Caregivers lose their everyday lives, disruption in their relationship with their spouses, difficulty in caring for other family members and discontinuation of family and social relationships.^23^ However, the caregivers feel obliged to care for their children despite its impact on the family routines and structure.^8^ Therefore, the caregivers of children with cancer need more support from the healthcare workers, family and community.

This study also showed that the participants were abandoned in the hospital and did not receive adequate social support from their families and communities. This is congruent with the findings of Bekui et al.^32^ in Ghana and those of Deribe et al.^30^ in Ethiopia, which showed that caregivers lacked support for the care of children left at home, household chores and farming. Malawi has only two paediatric cancer centres, and due to financial challenges, it may be challenging for the relatives of caregivers to visit them at these centres. This affects particularly low‐income families, especially those from rural areas,^30^ highlighting the need to strengthen institutional support for these caregivers. Furthermore, as suggested by Deribe et al.,^30^ there is a need to decentralise cancer treatment in Malawi so that people in remote areas of the country have equal access to care as those in central areas, which would also reduce the burden on caregivers and normalise some aspects of their lives.

It is important to know that the participants in this study experienced stigma associated with their child's cancer diagnosis from the family members and community. To avoid this hurtful experience, the caregivers chose to isolate themselves. Stigma and social isolation may reduce the mobilisation of social support that caregivers may require,^33^ thereby decreasing their ability to cope with the challenges of caring for a child with cancer. Furthermore, social isolation, regardless of the reason, can cause stress, anxiety and depression, and people need the support and stimulation that socialising provides.^34^, ^35^ Community education and awareness about cancer may help reduce cancer stigma and combat social isolation.^36^ When people are educated about cancer, they may change their attitude towards cancer patients and their caregivers.^33^ However, this is a new finding among the caregivers of children with cancer, which has never been reported elsewhere and thus requires further research.

Caregivers of children with cancer identified various coping skills to manage the challenges and stressors associated with child cancer diagnosis and caregiving. The most commonly used coping skills were religious practices, accepting the child's condition, social support, and information support which are supported by previous studies.^37^, ^38^, ^39^ Moreover, caregivers also used distractive behaviours, and this is congruent to the studies of caregivers of children with cancer.^37^, ^38^ Self‐distraction is considered a form of avoidance coping. While self‐distraction can help to deal with uncontrolled stressors,^40^ it may lead to anxiety and depressive symptoms if used for a prolonged period.^34^ Healthcare workers need to incorporate interventions to enhance the adaptive coping skills of caregivers of children with cancer in paediatric oncology care.

The findings of this study showed that the participants needed information on a child's cancer diagnosis, the recommended treatment and side effects, and ways to care for their child, which is congruent with the findings of other studies in caregivers of children with cancer.^31^, ^41^ However, caregivers in this current setting have unique informational needs, such as guidance on nutrition for the child with cancer and coping strategies for themselves. Providing caregivers with understandable information on paediatric cancer care that incorporates child nutrition and caregiver coping skills can help caregivers feel more in control of their child's condition, remain optimistic and participate effectively in their child's care.^31^, ^41^ This underscores the need for educational resources such as booklets, counselling and psychoeducation programmes to be made available to caregivers of children with cancer. Evidence has shown that education programmes are effective in improving the knowledge of caregivers and ensuring that caregivers understand the fundamental concepts involved in caring for a child with cancer.^42^

The study has found that Malawian caregivers have specific psychosocial needs that require attention and support. They stated that they would benefit from psychological support such as encouragement, empathy, interaction and sharing experiences with other caregivers of children with cancer, which is congruent with the other studies of caregivers of children with cancer conducted in Sri Lanka^31^ and Ethiopia.^22^ Malesee et al. reported that caregivers need psychological support, such as encouragement during the cancer trajectory for both themselves and their child.^22^ In Malawi, caregivers are vital in providing care and fulfilling essential caregiving responsibilities for their children.^13^ However, due to limited human resources, they often do not receive adequate psychological support and care.^29^ The development of psychosocial interventions for Malawian caregivers would be highly beneficial in supporting them throughout the child cancer journey, ultimately optimising their overall well‐being. In this regard, interventions such as group psychoeducation and support groups can offer peer support and increase the psychological support and guidance for these caregivers.^31^

Moreover, the caregivers in this study expressed the need for spiritual support, which aligns with the findings of Akaberia et al.^43^ Religious beliefs practised by the caregivers provided comfort that helped them cope with their child's illness. They suggested forming prayer groups, prayer schedules, and the support of the religious chaplain to strengthen their practice. Akaberia et al.^43^ found that the caregivers in Iran expressed the need for direct and indirect connectedness with God, asking God for help and trusting him. These religious beliefs help caregivers achieve peace and endure hardships and critical disease‐related situations. Literature reports that the absolute trust in God Almighty through religious practices buffers the emotional distress,^44^ enhances the acceptance of the child's condition and plays a role in coping with the child's illness.^43^, ^45^ In our study, the caregivers relied on their religious affiliations, and even though the religious chaplains were available, their operations seemed irregular and unclear. Furthermore, spiritual care is not prioritised in the healthcare system, and the caregivers never get individualised spiritual care.^32^ Hence, healthcare workers need to be aware of the spiritual needs of caregivers and offer them spiritual support throughout the cancer trajectory through appropriate interventions.

Moreover, the caregiving responsibilities had negatively impacted caregivers' source of income, and they struggled to meet the financial and material needs required to care for their child and other family members. This is in line with the previous studies, which showed that caregivers expressed the need for financial support due to high treatment costs, leaving their jobs and being unable to do business, which resulted in decreased income for caregivers.^22^, ^23^ Cancer is a chronic illness and requires long‐term treatment. Despite cancer treatment being free, most caregivers in the current setting experienced financial challenges related to transportation and food. Malawi is a low‐income country, and most of its population lives in rural areas where extensive poverty exacerbates financial difficulties. Therefore, there is a need for interventions that may help caution the caregivers financially as they are caring for their children with cancer.

### 4.1 Implications of the study

Caregivers of children with cancer in the current setting face myriad challenges that can significantly impact their ability to cope with their child's illness and provide care. As a result, healthcare workers in paediatric oncology units must assess and support the well‐being of caregivers and strive to meet their needs. A family‐centred approach to holistic care should be provided by healthcare workers involved in paediatric oncology. In light of this, protocols are necessary for providing support to caregivers and managers can utilise these findings to develop such protocols. Future research should use the findings of this study to develop ways to help caregivers of children with cancer, such as developing psychosocial interventions to meet their informational needs and reduce their psychological problems and offering strategies to help them cope with caregiving.

### 4.2 Limitations of qualitative study

This study has some limitations. First, the findings may not apply to a broader population of caregivers of children with cancer due to the small sample size. Furthermore, the majority of the participants were the caregivers of children with cancer within 6 months of diagnosis. Therefore, future studies should balance the number of participants across all the time spans. Another limitation is the inclusion of caregivers of children with different cancer types. Since the experience may differ based on the cancer type, future study needs to assess the experience of caregivers based on the type of cancer.

## 5 CONCLUSION

The results of this study offer valuable insights into the experiences of Malawian caregivers of children with cancer. The findings indicate that Malawian caregivers hold various perceptions of cancer, experience a lack of support, face unmet needs and use diverse strategies to cope with the challenges and stresses of caring for their child with cancer. Consequently, we recommend implementing measures like enhancing institutional support for caregivers and their children, decentralising cancer treatment to ensure equal access, developing interventions that can financially assist caregivers in their role, appointing a pastoral chaplain to oversee the spiritual welfare of caregivers in paediatric oncology units, establishing support groups for peer support to increase psychological support for these caregivers and developing educational programmes and community sensitisation. Therefore, policymakers can use these findings to develop effective interventions and protocols at a broader level.

## AUTHOR CONTRIBUTIONS

**Lophina Phiri:** Conceptualization (equal); data curation (lead); formal analysis (lead); methodology (lead); writing – original draft (lead); writing – review and editing (equal). **William Ho Cheung Li:** Conceptualization (equal); methodology (equal); supervision (lead); writing – review and editing (lead). **Patrick G. M. C. Phiri:** Data curation (equal); formal analysis (supporting); methodology (equal); writing – original draft (supporting); writing – review and editing (equal). **Ankie Tan Cheung:** Conceptualization (equal); methodology (equal); supervision (equal); writing – review and editing (equal). **Watipaso Kalizang'oma:** Data curation (equal); formal analysis (equal); methodology (equal); writing – review and editing (equal). **Anizia Kamwendo:** Data curation (equal); formal analysis (equal); methodology (equal); writing – review and editing (equal). **Sellina Lemon:** Data curation (equal); formal analysis (equal); methodology (equal); writing – review and editing (equal).

## CONFLICT OF INTEREST STATEMENT

The authors declare that they have no financial or non‐financial interest to disclose.

## Data Availability

The data that support the findings of this study are available from the corresponding author upon reasonable request.
